# Cardiovascular Risk Factors and Ethnicity Are Independent Factors Associated with Lower Urinary Tract Symptoms

**DOI:** 10.1371/journal.pone.0130820

**Published:** 2015-06-22

**Authors:** Jasmine Lim, Nirmala Bhoo-Pathy, Selvalingam Sothilingam, Rohan Malek, Murali Sundram, Guan Hee Tan, Badrulhisham Bahadzor, Teng Aik Ong, Keng Lim Ng, Azad Hassan Abdul Razack

**Affiliations:** 1 Department of Surgery, Faculty of Medicine, University of Malaya, Kuala Lumpur, Malaysia; 2 Julius Center University of Malaya, Department of Social and Preventive Medicine, Faculty of Medicine, University of Malaya, Kuala Lumpur, Malaysia; 3 Primary Care, University Medical Center Utrecht, 3508GA, Utrecht, Netherlands; 4 Department of Urology, Selayang Hospital, Selangor, Malaysia; 5 Department of Urology, Kuala Lumpur Hospital, Kuala Lumpur, Malaysia; 6 Department of Surgery, University Kebangsaan Malaysia Medical Center, Kuala Lumpur, Malaysia; 7 Centre for Kidney Disease Research, School of Medicine, University of Queensland, Translational Research Institute, Brisbane, Australia; Oklahoma University Health Sciences Center, UNITED STATES

## Abstract

**Objectives:**

To determine the lower urinary tract symptoms (LUTS) profile and factors affecting its degree of severity including cardiovascular risk profile, age, ethnicity, education level and prostate volume in a multiethnic Asian setting.

**Materials and Methods:**

We conducted a cross-sectional study of 1021 men aged 40–79 years with no clinical evidence of prostate cancer, prostate surgery or 5α-reductase inhibitor treatment of known prostate conditions. The severity of LUTS was assessed using the International Prostate Symptom Score (IPSS). Potential factors associated with LUTS including age, ethnicity, education, history of hypertension, diabetes and hypercholesterolemia, height, weight, and prostate volume were evaluated using univariable and multivariable analyses.

**Results:**

There were 506 (50%) men found to have moderate-to-severe LUTS attaining an IPSS above 7. Overall, nocturia (45.5%) was the most frequently reported symptom. Multivariable analysis showed that age, ethnicity, prostate volume and history of hypertension and hypercholesterolemia were independent factors associated with severity of LUTS (*p* < 0.05). Considering individual lower urinary tract symptoms, we found a strong association of storage symptom with history of hypertension and hypercholesterolemia. Malay men were significantly bothered by post micturition symptom compared to their Chinese and Indian counterparts. Stratified analyses of LUTS demonstrated a mutually exclusive cardiovascular risk factors profile defined by ethnicity.

**Conclusion:**

Severity of LUTS varies between different ethnicities across all age groups. In addition to age and prostate volume, ethnicity and cardiovascular risk factors including hypertension and hypercholesterolemia may also need to be taken into account in managing men with LUTS.

## Introduction

Lower urinary tract symptoms (LUTS) is subjective indicator of a disease or change in condition as perceived by the patient, partner or caregiver and may lead him / her to seek help from healthcare professionals. These symptoms are categorised into three groups including voiding symptoms (slow stream, splitting / spraying, intermittency, hesitancy, straining and terminal dribble), storage symptoms (increased daytime frequency, nocturia, urgency and urinary incontinence) and post micturition symptoms (feeling of incomplete emptying and post micturition dribble) [1]. The symptoms become increasingly common with age, impacting health-related quality of life [2].

The pathogenesis of LUTS remains to be fully elucidated. It has been hypothesised that LUTS may be attributed to endothelial dysfunction and pelvic atherosclerosis [3] that are closely linked to metabolic syndrome (MetS). Of note, MetS is a constellation of known cardiovascular risk factors including insulin resistance, obesity, atherogenic dyslipidemia and hypertension [4]. A high age-adjusted prevalence of MetS was observed in the U.S. population in which 22.9%–25.5% of adults being diagnosed with the syndrome from 1999 to 2010 based on the National Health and Nutrition Examination Survey [5]. Similar trend of MetS was recently reported in the multiethnic South East Asian population such as Malaysia (34.3%) [6] and Singapore (22.8%) [7] compared to other homogenous Asian populations in India (18.3%) [8], Hong Kong (9.6%) [9] and China (13.7%) [10].

There is emerging evidence suggesting associations between LUTS and MetS [11] or its components including obesity [12], hypertension and diabetes [13], although results have not been fairly consistent [14,15]. Therefore, it is of our interest to study the LUTS profiles amongst Asian men and improve the understanding of its potential link with MetS components. Malaysia is a high middle-income country in Southeast Asia with a multi-ethnic population encompassing mainly Malays, Chinese, Indians and indigenous races. We assessed the severity of LUTS across different age as well as ethnic groups and investigated the association of LUTS with age, ethnicity, education, anthropometric measurements (height, weight and prostate volume) and major comorbidities (diabetes, hypertension and hypercholesterolemia).

## Materials and Methods

### Study subjects

The current cross-sectional study was designed to recruit men above 40 years of age attending the prostate awareness campaign held in the Klang Valley, Malaysia in July 2011. There were eight participating hospitals in which five of them serve as tertiary referral centers for urology services in the region. Detailed methods of subject recruitment and data collection have been previously described [16].

A total of 1021 men were enrolled into the study. Clinical and demographic data such as age, ethnicity, education, height, weight and history of major comorbidities including diabetes, hypertension and hypercholesterolemia was ascertained using a structured questionnaire requiring input via face to face interviews, as well as medical examinations. LUTS were assessed using the validated IPSS (International Prostate Symptom Score) 7-item index [17]. All men underwent transrectal ultrasonography (TRUS) to determine the prostate volume. For assessment of voiding function, uroflowmetry was done to measure the maximum flow rate (Qmax) based on a minimum voided volume of 150 ml.

Each participant completed the interviews particularly the IPSS questionnaire and had no history of prostate cancer, previous history of prostate surgery or 5α-reductase inhibitor treatment of known prostate conditions. The baseline serum prostate specific antigen (PSA) level was measured in all subjects using total PSA assays described in [16]. Those of PSA > 4 ng/ml who refused for TRUS-guided prostate biopsy or confirmed with prostate cancer were excluded from the study. Overall, the serum PSA values ranged between 0.09–16.47 ng/ml.

All participants provided written informed consent. Ethical approval of this study was obtained from the medical research and ethics committee at the Ministry of Health Malaysia (code: NMRR-11-314-9262).

### Data analysis

We first determined the severity of LUTS according to IPSS [17] by age groups (40–49, 50–59, 60–69 and 70–79 years) as well as ethnicity (Malay, Chinese and Indian). Lower urinary tract symptoms were further characterised into three groups; storage (frequency, nocturia & urgency), voiding (slow stream, intermittency & straining) and post micturition (incomplete emptying) symptoms. The ethnic heterogeneity of Qmax was examined using Kruskal-Wallis test.

Based on the severity of LUTS, participants were grouped into two categories using IPSS score > 7 as a cut-off point. Factors associated with LUTS including age, ethnicity, education, history of major comorbidities (diabetes, hypertension and hypercholesterolemia) and anthropometric variables (height, weight and prostate volume) were compared between two groups. We used univariable and multivariable logistic regression to study the associations of these variables with the severity of LUTS. The multivariable model was then used to explore potential clinical and demographic variables associated with storage, voiding or post micturition symptoms. In this analysis, the voiding and storage symptoms were dichotomised using a cut-off score of 3 whilst post micturition symptoms was defined with incomplete emptying score > 1.

In order to assess whether the association between ethnicity and severity of LUTS was modified by major comorbidities (diabetes, hypertension and hypercholesterolemia), likelihood ratio tests were applied between nested models with and without multiplicative interaction terms. The missing data in most of the variables was less than 0.5% except education level and prostate volume with around 6% and 25% respectively. This was expected owing to the more sensitive nature of socioeconomic status and invasive approach of measuring prostate volume using transrectal ultrasonography (TRUS). All statistical analysis was performed using SPSS for Windows version 21.0 (SPSS Inc., Chicago, Illinois, USA). Two-tailed *P* value < 0.05 was termed as statistically significant.

## Results

There were 1021 men participated in the study and the median age was 59 years, ranging from 40 to 79 years. Most of the participants were Chinese (509; 49.9%), followed by Malays (367; 35.9%) and Indians (145; 14.2%). In term of major comorbidities, the proportions of subjects with history of hypercholesterolemia, diabetes and hypertension were 18.3%, 24.5% and 38.6% respectively.

In this study, 95% of subjects reported some degree of LUTS, of whom approximately half of the subjects reported moderate-to-severe LUTS (IPSS > 7), with a higher prevalence in men aged 70–79 years than those of 50–59 years (64% versus 43%) (Table 1). Differences in LUTS reporting were observed across various ethnicities in which Malay men (55%) were more likely to have moderate-to-severe LUTS symptoms compared to Chinese (48%) and Indians (42%). Interestingly, the median maximum flow rate (Qmax) of Malay, Chinese and Indian was 13.1 ml/s, 13.9 ml/s and 13.6 ml/s respectively (*p* = 0.158; Kruskal Wallis test), indicating that there is no association between ethnicity and Qmax. In addition, majority of individual lower urinary tract symptom became more prevalent with increasing age. Nocturia was the symptom (45.5%) most frequently found amongst all participants (Table 1).

**Table 1:** The LUTS scores by age group and ethnicity.

	Age group (years)				Ethnicity			
	40–49	50–59	60–69	70–79	Malay	Chinese	Indian	Total
**IPSS**								
None	6	27	17	3	22	19	12	53
(0)	(8.3%)	(5.7%)	(4.5%)	(3.3%)	(6.0%)	(3.7%)	(8.3%)	(5.2%)
Mild	28	244	161	29	144	246	72	462
(0–7)	(38.9%)	(51.1%)	(42.1%)	(32.2%)	(39.2%)	(48.3%)	(49.7%)	(45.2%)
Moderate	30	162	156	42	152	192	46	390
(8–19)	(41.7%)	(34.0%)	(40.8%)	(46.7%)	(41.4%)	(37.7%)	(31.7%)	(38.2%)
Severe	8	44	48	16	49	52	15	116
(20–35)	(11.1%)	(9.2%)	(12.6%)	(17.8%)	(13.4%)	(10.2%)	(10.3%)	(11.4%)
**Total**	72	477	382	90	367	509	145	1021
	(7.1%)	(46.7%)	(37.4%)	(8.8%)	(35.9%)	(49.9%)	(14.2%)	(100%)
**Storage symptoms**								
Frequency^	32	188	150	40	155	201	54	410
	(44.4%)	(39.4%)	(39.3%)	(44.4%)	(42.2%)	(39.5%)	(37.2%)	(40.2%)
Urgency^	19	107	113	37	109	122	45	276
	(26.4%)	(22.4%)	(29.6%)	(41.1%)	(29.7%)	(24.0%)	(31.0%)	(27.0%)
Nocturia^†^	27	181	197	60	170	231	64	465
	(37.5%)	(37.9%)	(51.6%)	(66.7%)	(46.3%)	(45.4%)	(44.1%)	(45.5%)
**Voiding symptoms**								
Intermittency^	24	140	131	40	140	160	35	335
	(33.3%)	(29.4%)	(34.3%)	(44.4%)	(38.1%)	(31.4%)	(24.1%)	(32.8%)
Weak stream^	29	161	152	45	152	189	46	387
	(40.3%)	(33.8%)	(39.8%)	(50%)	(41.4%)	(37.1%)	(31.7%)	(37.9%)
Straining^	15	87	78	23	75	103	25	203
	(20.8%)	(18.2%)	(20.4%)	(25.6%)	(20.4%)	(20.2%)	(17.2%)	(19.9%)
**Postmicturition symptoms**								
Incomplete^ emptying	31	179	157	43	182	177	51	410
	(43.1%)	(37.5%)	(41.1%)	(47.8%)	(49.6%)	(34.8%)	(35.2%)	(40.2%)

^†^ Nocturia was defined as having more than one episode per night.

^ Subjects reporting the respective conditions with more than 1 time in 5 (score > 1) were recorded as having the symptoms.

**Fig 1:**
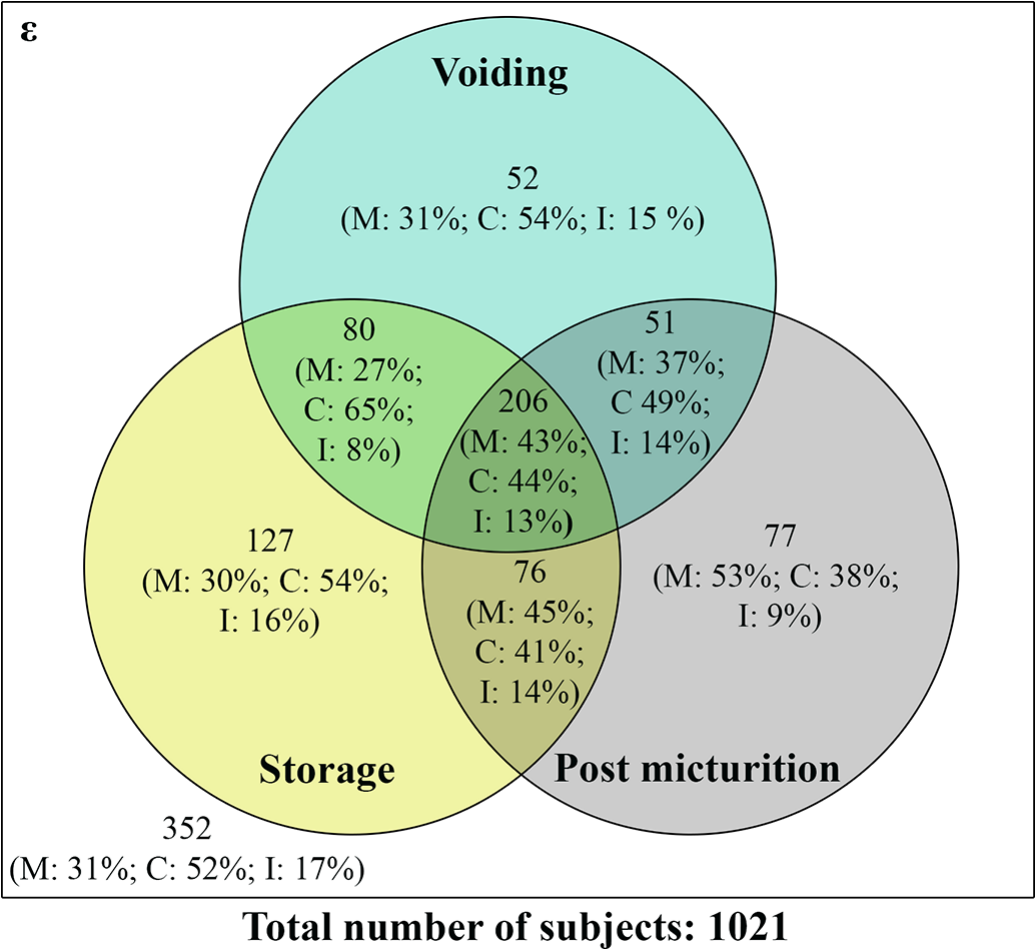
Profile of storage, voiding and post micturition symptoms amongst various ethnicities. Venn diagram showing the distribution of urological symptoms in Malay (M), Chinese (C) and Indian (I) participants.

Comparing LUTS in three clusters of urinary symptoms (Fig 1), storage scores were documented in approximately half of the individuals (489; 48%) and amongst these, 206 (42%) were bothered with both voiding and post micturition symptoms. Overall, 256 (25%) men had storage, voiding or post micturition symptoms only whilst combinations of these symptoms were observed in 413 (40%) subjects. For post micturition symptoms alone, nearly half of the Malay men (~ 53%) presented with the symptoms characterised by incomplete emptying (Fig 1). Chinese (44%) and Malays (43%) were more likely to have all three symptoms compared to their Indian counterparts (13%) (Fig 1).

Age, ethnicity, education, history of hypertension, diabetes and hypercholesterolemia as well as prostate volume were found to be significantly associated with moderate-to-severe LUTS (Table 2). For instance, participants with history of hypertension or hypercholesterolemia were 1.6-fold more likely than those without these medical conditions to have moderate-to-severe LUTS (*p* < 0.01). With every 1 year increase in age, there was a 1% rise in the probability of reporting moderate-to-severe LUTS (*p* = 0.000). We did not observe any significant associations between LUTS and height or weight. In the multivariable analysis, age, ethnicity, history of hypertension and hypercholesterolemia as well as prostate volume remained significantly and independently associated with moderate-to-severe LUTS (Table 3).

**Table 2:** Comparison of factors associated with LUTS.

	Frequency distribution						
	Mild LUTS (IPSS ≤ 7)		Moderate to severe LUTS (IPSS > 7)				
Factors	No.	%	No.	%	OR	95% CI	*P*
**Age**	515	50.4	506	49.6	1.04	1.02–1.05	*0*.*000*
Median	58		60				
**Ethnicity**							
Malay	166	32.2	201	39.7	1.00		
Chinese	265	51.5	244	48.2	0.76	0.58–1.00	*0*.*046*
Indian	84	16.3	61	12.1	0.60	0.41–0.89	*0*.*010*
**Education**							
Primary	65	13.4	89	18.9	1.00		
Secondary	253	52.3	227	48.2	0.66	0.45–0.95	*0*.*024*
Tertiary	166	34.3	155	32.9	0.68	0.46–1.01	0.053
*unknown	31		35				
**Diabetes**							
No	405	78.6	366	72.3	1.00		
Yes	110	21.4	140	27.7	1.41	1.06–1.88	*0*.*019*
**Hypertension**							
No	344	66.8	283	55.9	1.00		
Yes	171	33.2	223	44.1	1.59	1.23–2.04	*0*.*000*
**Hypercholesterolemia**							
No	437	84.9	397	78.5	1.00		
Yes	78	15.1	109	21.5	1.54	1.12–2.12	*0*.*009*
**Height (m)**	514	50.5	503	49.5	0.99	0.97–1.00	0.137
Median	166		165				
*unknown	1		3				
**Weight (kg)**	514	50.5	503	49.5	1.00	0.99–1.01	0.953
Median	70		69				
*unknown	1		3				
**Prostate volume (ml)**							
< 30	264	69.7	229	59.5	1.00		
≥ 30	115	30.3	156	40.5	1.56	1.16–2.11	*0*.*003*
*unknown	136		121				

Abbreviations: CI, confidence interval; IPSS, international prostate symptom score; LUTS, lower urinary tract voding symptoms; OR, odds ratio.

**Table 3:** Multivariable analysis of factors associated with LUTS.

Factors	Regression coefficient*	OR	95% CI	*P*
**Age (year)** ^†^	0.029	1.03	1.01–1.05	*0*.*002*
**Ethnicity**				
Malay	Baseline			
Chinese	-0.364	0.70	0.52–0.93	*0*.*015*
Indian	-0.578	0.56	0.37–0.84	*0*.*005*
**Education**				
Primary	Baseline			
Secondary	-0.333	0.72	0.49–1.05	0.089
Tertiary	-0.259	0.77	0.51–1.16	0.213
**Diabetes**				
No	Baseline			
Yes	0.191	1.21	0.88–1.66	0.240
**Hypertension**				
No	Baseline			
Yes	0.304	1.36	1.02–1.80	*0*.*035*
**Hypercholesterolemia**				
No	Baseline			
Yes	0.340	1.41	1.00–1.97	*0*.*049*
**Height (m)** ^†^	-0.005	1.00	0.98–1.01	0.552
**Weight (kg)** ^†^	-0.004	1.00	0.99–1.01	0.492
**Prostate volume**				
< 30	Baseline			
≥ 30	0.374	1.45	1.06–1.99	*0*.*019*

*Multivariate model inlcudes age, ethnicity, education level, history of diabetes, hypertension & hypercholesterolemia as well as prostate volume.

^†^ Age (year), height (m) and weight (kg) considered as continuous variable within the multivariate model.

Abbreviations: CI, confidence interval; OR, odds ratio.

We then stratified the multivariable analysis according to ethnicity. Intriguingly, a mutually exclusive association pattern was observed between severity of LUTS and cardiovascular risk factors. For Indian, history of diabetes (adjusted OR 2.69, 95% CI 1.17–6.19) and hypercholesterolemia (adjusted OR 3.24, 95% CI 1.11–9.46) were strongly associated with moderate-to-severe LUTS whilst hypertension (adjusted OR 1.70, 95% CI 1.13–2.56) was found significantly related with severity of LUTS in Chinese only. Conversely, there were no association between cardiovascular risk profiles and LUTS in Malay. Collectively, cardiovascular risk factors did not seem to modify the association between ethnicity and LUTS: history of diabetes (*P*_interaction_ = 0.312), hypertension (*P*_interaction_ = 0.194) and hypercholesterolemia (*P*_interaction_ = 0.349).

The multivariable model was further tested in three categories of LUTS consisting of storage, voiding and post micturition symptoms as summarised in Table 4. Malay ethnicity and history of hypertension were associated with post micturition symptoms. Age and components of cardiovascular risk factors including history of hypercholesterolemia as well as hypertension were independent factors of increased storage symptoms. For voiding symptoms, subjects with a prostate volume ≥ 30 ml was 1.4-fold more likely than those of a lower prostate volume to present with a voiding score > 3 (*p* = 0.043), in addition to age. Both post micturition and voiding symptoms were ~ 60% more likely to be found in men with lower education than those of tertiary education level.

**Table 4:** Multivariate analysis of factors associated with post micturition, voiding and storage symptoms.

	Post micturition score > 1	Voiding score > 3	Storage score > 3
Factors	OR (95% CI); *P* value		
**Age (year)** ^†^	1.01 (0.99–1.03); 0.318	1.02 (1.01–1.04); *0*.*015*	1.04 (1.02–1.05); *0*.*000*
**Ethnicity**			
Malay	1.00	1.00	1.00
Chinese	0.49 (0.36–0.66); *0*.*000*	0.89 (0.66–1.20); 0.440	0.85 (0.64–1.14); 0.289
Indian	0.53 (0.35–0.80); *0*.*003*	0.72 (0.47–1.10); 0.126	0.74 (0.49–1.11); 0.141
**Education**			
Primary	1.00	1.00	1.00
Secondary	0.62 (0.43–0.91); *0*.*016*	0.70 (0.48–1.03); 0.071	0.83 (0.57–1.23); 0.345
Tertiary	0.57 (0.38–0.86); *0*.*008*	0.62 (0.41–0.93); *0*.*021*	0.86 (0.57–1.29); 0.471
**Diabetes**			
No	1.00	1.00	1.00
Yes	1.16 (0.84–1.59); 0.374	1.26 (0.92–1.74); 0.157	1.02 (0.74–1.40); 0.895
**Hypertension**			
No	1.00	1.00	1.00
Yes	1.36 (1.02–1.81); *0*.*038*	1.00 (0.75–1.33); 0.981	1.51 (1.14–2.00); *0*.*004*
**Hypercholesterolemia**			
No	1.00	1.00	1.00
Yes	0.90 (0.64–1.27); 0.559	1.37 (0.98–1.91); 0.071	1.55 (1.11–2.17); *0*.*011*
**Height (m)** ^†^	0.99 (0.97–1.01); 0.292	1.00 (0.98–1.01); 0.584	1.00 (0.98–1.02); 0.969
**Weight (kg)** ^†^	0.99 (0.98–1.00); 0.106	1.00 (0.98–1.01); 0.455	1.00 (0.99–1.01); 0.791
**Prostate volume (ml)**			
< 30	1.00	1.00	1.00
≥ 30	1.01 (0.74–1.39); 0.947	1.39 (1.01–1.90); *0*.*042*	1.30 (0.95–1.78); 0.096

^†^ Age (year), height (m) and weight (kg) considered as continuous variables within the multivariate model.

Abbreviations: CI, confidence interval; OR, odds ratio.

## Discussion

Findings from this study provide an insight into the severity of LUTS in a large cohort of multiethnic Asian men. We demonstrated that there were significant ethnic variations in the prevalence of LUTS particularly in post micturition symptoms. Major elements of metabolic syndrome especially hypercholesterolemia and hypertension were significantly associated with LUTS.

LUTS are often attributed to the benign prostatic hyperplasia (prostate), detrusor overactivity-overactive bladder syndrome (bladder) or nocturnal polyuria (kidney) [18]. The population-based Olmsted County cohort study convincingly demonstrated an increase in LUTS over time [19]. Here, we revealed that age was significantly associated with moderate-to-severe LUTS. The age-dependent LUTS trend was consistently observed in previous multiethnic Asian population-based cross-sectional studies [20–22]. It is worth noting that the prevalence of the current study (49.6%, Table 1) is much higher than those previously reported ranged 6–29% [20–22], owing to different population sampling methods. Comparing to the randomised / multistage sampling technique adopted in previous reports [20–22], the nature of current study design might hamper the sample by a selection bias although our aim was not to calculate prevalence rates.

To our knowledge, this is the first study which found ethnicity as an independent factor associated with LUTS particularly for post micturition symptom. Our results identified that Malay men were significantly more bothered with LUTS related to incomplete emptying and post micturition dribble than their Chinese and Indian counterparts. This could be attributed to the religious practice of most Malay as Muslims. It is part of their rituals to perform washing (*wudhu*) and be clean prior to performing the daily prayers. Therefore, post micturition symptoms have a greater impact on Malay (Muslim) men compared to other non-Muslim ethnicities. It is noteworthy that potential dietary and genetic factors which might explain fundamental differences in the presentation of urinary symptoms across various ethnicities remain under investigations. In addition, further analysis showed that there was no significant difference of median Qmax across different ethnicities suggesting that management of LUTS patients should be personalised based on the severity of storage, voiding and post micturition symptoms. This is because current treatment of LUTS with alpha-blocker monotherapy may improve one’s Qmax and benefits patients with storage or voiding symptoms only.

Our finding showed that a high prostate volume (e.g. ≥ 30 ml) was significantly associated with LUTS particularly in voiding symptoms, indicating that LUTS patients affected predominantly with voiding symptoms are likely to have a bigger prostate compared to those bothered by other urinary symptoms. Roehrborn *et al* [23] analysed long term symptoms changes in relation to prostate volume amongst the placebo-treated men with moderate-to-severe LUTS for 4 years in the Proscar Long Term Efficacy and Safety Study and determined that high baseline enlarged prostate volume predicted deterioration of LUTS over time. This is further supported by results from the Medical Therapy of Prostatic Symptoms trial placebo group showing baseline prostate volume correlated significantly with the American Urological Association symptom index score [24].

Increased post micturition or voiding score was linked with education level. This is consistent with the epidemiological dogma in which a low education level or socioeconomic status is linked to more disease of any kind. The discrepancy also suggests that men are more aware and could tolerate better with storage symptoms compared to post micturition and voiding symptoms, regardless of the education level.

Furthermore, we observed an independent association between LUTS and cardiovascular risk factors including history of hypertension and hypercholesterolemia, especially in storage and post micturition symptoms. The association between LUTS and cardiovascular risk factors has been previously documented [13,20,25]; nevertheless, several of our findings are novel particularly those associated with specific urinary symptoms groups. For instance, metabolic syndrome [11] or a low level of high density lipoprotein [26] was previously associated with voiding symptoms only. Results from the present study add to the growing literature that underlying medical illness (i.e. hypertension and hypercholesterolemia) may also impact in storage and post micturition symptoms. Previous studies proposed that pelvic atherosclerosis specifically ischemia and endothelial dysfunction, which contributed to decreased nitric oxide (NO) bioavailability, were potential entities linking between MetS and LUTS. Notably, NO is crucial in maintaining vascular health by inhibiting adhesion of platelets and leukocytes to the vascular wall as well as decreasing proliferation of vascular smooth muscle which may result in initiation of atherosclerosis [27]. Under the low NO levels in MetS, excessive RHO-kinase (ROK) activation can result in greater tonic prostatic smooth muscle contraction contributing to LUTS [28]. In addition, pre-clinical data using hyperlipidemic rats model demonstrated that prostatic enlargement and bladder overactivity were consistently observed in rats with high serum cholesterol and low density lipoprotein [29], indicating the role of cardiovascular risk factors in promoting LUTS.

Our study findings provide a new understanding of LUTS profiles according to storage, voiding and post micturition symptoms within a multiethnic Asian population. This is also the first documented evidence of ethnicity as an independent factor associated with moderate-to-severe LUTS particularly in post micturition symptoms. The study highlighted the significant association between LUTS and cardiovascular risk profile including history of hypertension and hypercholesterolemia, in addition to age and prostate volume. Increased odds of hypercholesterolemia and hypertension were described with storage scores > 3. With the recognition of association between cardiovascular risk factors and urologic symptoms, we would like to recommend that clinicians should consider screening patients presenting with urologic symptoms for cardiovascular risk factors. Similarly, the LUTS-IPSS index should be assessed whilst planning treatment for patients with hypertension and hypercholesterolemia. Another important clinical implication of this study is that ethnicity and severity of different groups of urological symptoms may also need to be taken into account in addressing the management of LUTS, in addition to age and prostate volume.
